# Lack of hydraulic recovery as a cause of post‐drought foliage reduction and canopy decline in European beech

**DOI:** 10.1111/nph.18065

**Published:** 2022-03-23

**Authors:** Matthias Arend, Roman Mathias Link, Cedric Zahnd, Günter Hoch, Bernhard Schuldt, Ansgar Kahmen

**Affiliations:** ^1^ Physiological Plant Ecology University of Basel 4056 Basel Switzerland; ^2^ Ecophysiology and Vegetation Ecology Universität Würzburg 97082 Würzburg Germany

**Keywords:** canopy dieback, drought legacy, *Fagus sylvatica*, leaf development, leaf to sapwood area, loss of xylem hydraulic conductance, nonstructural carbohydrates

## Abstract

- European beech (*Fagus sylvatica*) was among the most affected tree species during the severe 2018 European drought. It not only suffered from instant physiological stress but also showed severe symptoms of defoliation and canopy decline in the following year.
- To explore the underlying mechanisms, we used the Swiss‐Canopy‐Crane II site and studied in branches of healthy and symptomatic trees the repair of hydraulic function and concentration of carbohydrates during the 2018 drought and in 2019.
- We found loss of hydraulic conductance in 2018, which did not recover in 2019 in trees that developed defoliation symptoms in the year after drought. Reduced branch foliation in symptomatic trees was associated with a gradual decline in wood starch concentration throughout summer 2019. Visualization of water transport in healthy and symptomatic branches in the year after the drought confirmed the close relationship between xylem functionality and supported branch leaf area.
- Our findings showed that embolized xylem does not regain function in the season following a drought and that sustained branch hydraulic dysfunction is counterbalanced by the reduction in supported leaf area. It suggests acclimation of leaf development after drought to mitigate disturbances in canopy hydraulic function.

## Introduction

Severe drought has long been recognized as a serious environmental threat to forest ecosystems and as a primary cause of globally observed forest health decline and tree mortality (Allen *et al*., 2010, 2015; Hartmann *et al*., 2018). Over the last two decades, several unprecedented dry spells impacted forests around the globe, causing large shifts in the structure and function of forest ecosystems (Ciais *et al*., 2005; Gharun *et al*., 2020; Schuldt *et al*., 2020; Büntgen *et al*., 2021). This has prompted intense research on the physiological nature of drought‐induced forest decline and tree mortality (Adams *et al*., 2017; Choat *et al*., 2018). Still, our knowledge of mechanisms triggering tree damages and mortality under severe drought is far from complete, leaving large uncertainty in physiological tree and vegetation models predicting the fate of forests under climate change (McDowell *et al*., 2022).

To date, two interconnected concepts attempt to explain physiological pathways to drought‐induced health decline and tree mortality. These are catastrophic failure of hydraulic water transport due to excessive embolism formation on the one hand, and disorders of photosynthesis and subsequent carbon starvation on the other hand (McDowell *et al*., 2008, 2011). Due to methodological constraints, most of the current knowledge in these fields originates from observations on small tree saplings exposed to artificially induced droughts (e.g. Hartmann *et al*., 2013a,b; Urli *et al*., 2013; Hammond *et al*., 2019), while similar observations of *in situ* xylem functionality and carbon status in mature, field‐grown trees experiencing natural droughts are relatively rare in the scientific literature. Recent field observations on mature spruce, poplar, beech and eucalypt trees, however, provided unequivocal evidence for hydraulic failure as a universal cause of defoliation, crown dieback and eventually mortality under natural drought in these genera (Anderegg *et al*., 2012; Arend *et al*., 2021; Nolan *et al*., 2021; Walthert *et al*., 2021). Importantly, drought‐induced embolism may not necessarily lead to severe damage and mortality if a tree is able to repair hydraulic dysfunction before critical levels of tissue dehydration and cellular death are reached. To date, it remains unclear whether and to what extent mature trees can activate repair mechanisms to restore hydraulic function after a natural drought (Sperry, 2013; Charrier *et al*., 2016; Klein *et al*., 2018; Trifilò *et al*., 2019).

A limitation in current approaches to explain drought‐induced tree decline may arise from very variable die‐off patterns that tall trees may show in response to natural drought. These can range from rapid whole‐tree death to slowly progressing leaf area loss, partial branch dieback and eventually tree death in subsequent years. The latter situation appears to be more common than the current mechanistic frameworks of tree mortality anticipate. In fact, national forest monitoring data and crown vitality assessments show often lagged responses of trees to severe drought with striking leaf area reductions and increasing crone transparency in the years following the stress (Solberg, 2004; UN/ECE, 2005; Bréda *et al*., 2006; Seidling, 2007). In beech, for example, such symptoms were observed up to 3 yr after release from drought (Seidling, 2007). It demonstrates that the damage of a tree must not necessarily be considered as an instant response to drought stress, but it may also include long‐lasting drought legacies on crown development, similar to drought legacies observed in tree ring studies (Anderegg *et al*., 2015). Yet, it remains unclear whether such legacies just reflect the lagged expression of a physical injury or a physiologically coordinated post‐drought acclimation response that prepares a tree for recurrent droughts (Gessler *et al*., 2020).

In this study, we investigated the physiological consequences of the severe 2018 central European drought event that caused widespread damages to forests, with widespread symptoms of crown defoliation and partial branch dieback in European beech in the following year (Schuldt *et al*., 2020). Specifically, we tested: (1) whether branch hydraulic conductance recovered over the winter or with the start of the new growing season; (2) whether branch hydraulic dysfunction and recovery were associated with post‐drought canopy health conditions; and (3) whether branch carbohydrate reserves did change during and after drought. To do so, we used the unique instrumentation of the Swiss‐Canopy‐Crane II site to access the upper canopies of mature beech trees (*Fagus sylvatica*). We assessed canopy health conditions (i.e. occurrence of branches with reduced leaf development or dead branches) in selected trees and followed the native loss of xylem hydraulic conductance and variations in the concentration of nonstructural carbohydrates in upper canopy branches in the 2 yr during and after drought.

## Materials and Methods

### Research site

All research was done at the Swiss‐Canopy‐Crane II (SCCII) research site close to Hölstein/BL, Switzerland (47°26′17″N, 7°46′37″E; 550 m above sea level). The site is situated in a semi‐natural, uneven‐aged forest of the eastern Swiss Jura Mountains, with European beech (*F. sylvatica* L.) and Norway spruce (*Picea abies* L.) as dominant tree species and overall stand density of 46.3 m^2^ ha^−1^. The soil is characterized by high clay content (≥ 40%) and varying inclusions of calcareous rocky material from the underlying bedrock. The average regional climate conditions are 9°C annual temperature and 1009 mm annual precipitation (data taken from MeteoSwiss; https://www.meteoswiss.admin.ch/). The technical infrastructure includes 24 automated soil moisture probes (ML3 ThetaProbe; Delta‐T Devices Ltd, Burwell, UK) installed at eight locations across the site at 40‐cm soil depth; a weather station (Davis Vantage Pro2; Davis Instruments Corp., Hayward, CA, USA), installed 2 m aboveground in a forest gap; and a canopy crane with a height of 45 m and a crane radius of 50 m.

### Climatic conditions

**Fig. 1:**
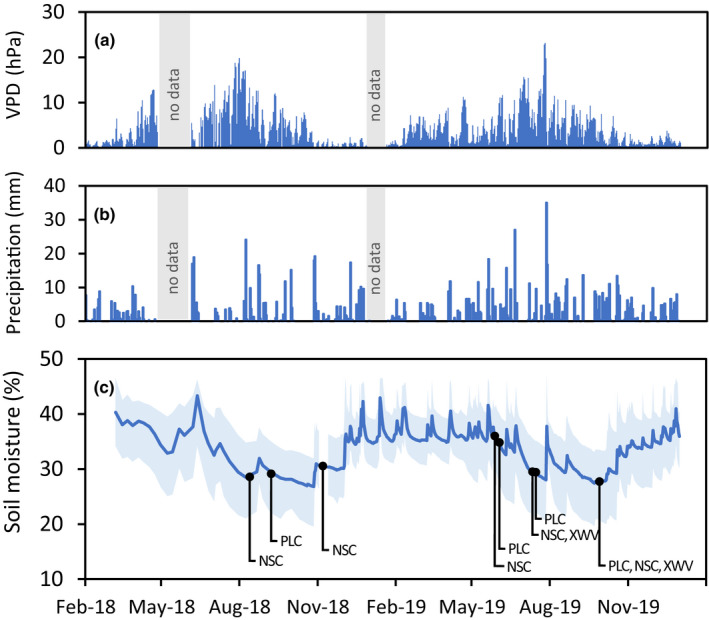
Seasonal weather and soil moisture conditions throughout the dry year 2018 and the following year 2019. (a) Daily vapour pressure deficit (VPD; average 00:00 h to 23:00 h CET), (b) daily precipitation and (c) soil moisture at 40‐cm soil depth (solid line, average of 8 measurement positions across the site; shaded area, min./max. variation across the 8 measurement positions). All sampling dates for analysis of native loss of xylem hydraulic conductance (PLC) and nonstructural carbohydrates (NSC), as well as for xylem water transport visualization (XWV), indicated by vertical lines (PLC, 4‐Sep‐2018/5‐Jun‐2019/11‐Jul‐2019/5‐Sep‐2019; NSC, 7‐Aug‐2018/5‐Nov‐2018/28‐May‐2019/9‐Jul‐2019/5‐Sep‐2019; XWV, 9‐Jul‐2019/5‐Sep‐2019).

The climatic conditions in the two study years were characterized by a severe drought during summer and autumn 2018 with high vapour pressure deficits and low precipitation (Hari *et al*., 2020), followed by a wet winter and spring and a moderate dry summer in 2019 with heavy intermediate rainfall (Fig. 1a,b). The climatic drought resulted in high hydraulic stress on the clay‐rich soil, with soil moisture declining on average to 27% in the upper soil (40 cm) (Fig. 1c), and minimum water potentials in the beech trees coming close to or even exceeding the P_12_ limit to the onset of excessive embolism formation (Supporting Information Table S1). Notably, not all individual trees experienced the same level of hydraulic stress during the 2018 drought, likely due to local variations of remaining soil moisture (Fig. 1c) and differences in the accessible soil volume across the site.

### Assessment of canopy health conditions

**Fig. 2:**
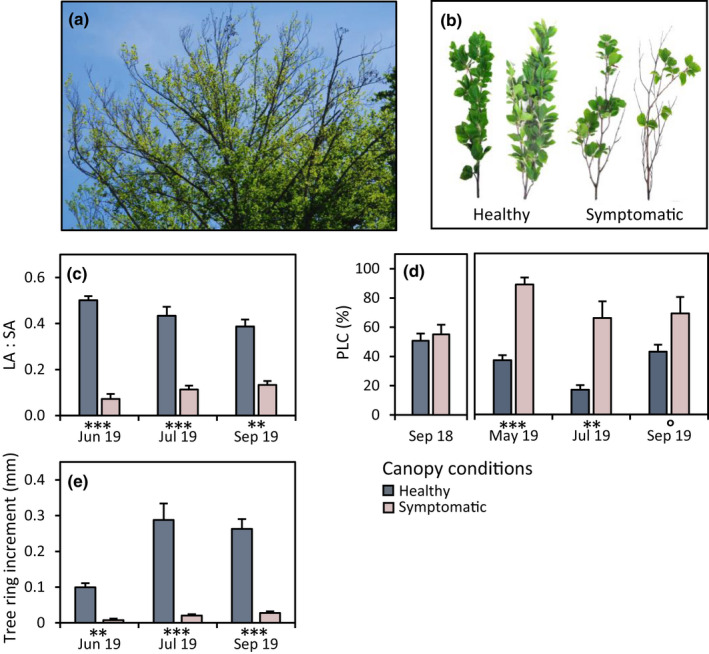
Canopy health decline in beech trees in the year after the severe 2018 drought and native percentage loss of hydraulic conductance (PLC), foliage reduction and tree ring increment in upper canopy branches. (a) Sparse foliage development and partial branch drying in the upper canopy in late spring 2019. (b) Branches collected from the upper part of healthy and symptomatic beech canopies. (c) Degree of foliation of upper canopy branches (LA : SA; total leaf area (LA) normalized to sapwood area (SA)) in trees becoming symptomatic or remaining healthy in 2019. (d) PLC values in upper canopy branches of the studied beech trees under severe drought in 2018 (bars show the average of trees remaining healthy or becoming symptomatic in the following vegetation season 2019) and in healthy and symptomatic canopies in the following summer half‐year 2019. (e) Tree ring increment in summer half‐year 2019 in upper canopy branches (data are mean ± SE, *n* = 5; *post hoc* tests for differences between groups with: ***, *P* ≤ 0.001; **, *P* ≤ 0.01; ᵒ, *P* ≤ 0.1; a small subset of data shown in (c) and (d) is included in Schuldt *et al*., 2020).

Beech canopies were visually inspected after the completion of leaf unfolding in late spring 2019 using the canopy crane. At this time, many mature tree individuals developed distinct decline symptoms in their upper canopies, which were not visible in the preceding drought year, 2018. Trees with arrested buds, sparse foliage development and partial branch drying in their upper canopy (Fig. 2a,b) were categorized as ‘symptomatic’, while trees without obvious signs of these post‐drought symptoms were categorized as ‘healthy’. In the following analysis, the two categories of trees were compared with each other to elaborate the mechanisms underlying the observed post‐drought symptoms of canopy decline.

### Branch sampling

For the analysis of percentage loss of hydraulic conductance (PLC), degree of foliation and nonstructural carbohydrates (NSC), we selected branches from 10 mature trees within the canopy crane radius: five being symptomatic in spring 2019 and five without visible symptoms as reference. The selected trees had a height between 26 and 30 m and a breast height diameter ranging from 38 to 61 cm. All trees had their upper canopies exposed to the free, turbulent atmosphere. The branch sampling was done at three dates during summer half‐year 2019 for each of the studied parameters (Fig. 1c). Branches for xylem water transport visualization were collected from the same trees at two dates in 2019 (Fig. 1c). For our measurements, all branches were taken from upper, sun‐exposed canopies in the morning hours using the canopy crane and immediately processed for further analysis.

To compare the 2019 physiological status of the trees with the actual status during the 2018 drought, we included PLC and NSC data in our analysis that were collected independently from 10 mature trees in 2018 on one date during the severe drought and one additional date (for NSC) after drought release (Fig. 1c). These trees did not show symptoms of canopy decline when samples were collected in 2018 but were retrospectively categorized as remaining ‘healthy’ and becoming ‘symptomatic’ in 2019. As sampling campaigns in 2018 and 2019 were independent of each other, we ended up analysing some trees selected in 2018 that we did not assess in 2019 and vice versa.

### Analysis of percentage loss of hydraulic conductance

Branch samples of a length of *c.* 100 cm were collected from the upper canopy of the 10 studied beech trees, exceeding the maximum vessel length of 20 cm reported for this species (Lübbe *et al*., 2022). The branch samples were immediately stored in dark humidified plastic bags. During the measurement campaign in early September 2018, two branches per tree were selected and measured on the same day in the laboratory of the University of Basel. In all subsequent campaigns, one branch per tree was selected and initial measurements likewise performed on the same day after transporting the samples to the University of Würzburg. Before measurement, samples were recut under water at least two times, and therefore, a total segment of at least 1.5 times maximum vessel length was dismissed. The segment was left submerged with its basipetal ends for 20 min to relax xylem tension and avoid artefacts induced by cutting under tension (Torres‐Ruiz *et al*., 2015). Subsequently, an unbranched segment of an average length of 64.7 ± 11.2 (SD) mm and an average diameter of 7.1 ± 1.1 (SD) mm was excised with a razor blade, debarked at the basipetal end and connected to a Xyl'Em‐Plus embolism meter (Bronkhorst France, Montigny‐les‐Cormeilles, France) using silicone tubing. Conductance measurements were performed submerged in water with a low‐pressure head of *c.* 1.5 kPa to avoid displacing emboli and using measurement solution of 10 mM KCl and 1 mM CaCl_2_ in degassed ultrapure water filtered to a particle size of 0.2 µm. After measuring the initial conductance (*K*_i_), the samples were rehydrated in measurement solution for at least 48 h under a partial vacuum before measuring saturated conductance (*K*_f_). The per cent loss of conductance (PLC, %) was then calculated as PLC = 100⋅(1−*K*_i_/*K*_f_) based on readings from the XylWin 3.0 software (Bronkhorst, Montigny‐les‐Cormeilles, France).

### Leaf area measurement

The total leaf area supported by the collected branch sample used for PLC measurements was calculated from A3 scans obtained with a flatbed scanner (Epson Expression 12000XL; Epson, Nagano, Japan) using ImageJ v.1.52a (https://imagej.nih.gov/ij/) and expressed as leaf‐to‐sapwood area ratio (m^2^ cm^−2^).

### Analysis of nonstructural carbohydrates

Concentrations of nonstructural carbohydrates (starch and the low molecular weight carbohydrates glucose, fructose and sucrose) were measured in leaves, bark and wood of the sampled branches. The enzymatic–photometric method was used as previously described in detail (Landhäusser *et al*., 2018). In short, low molecular weight sugars were extracted from plant powder with 80% ethanol at 90°C. After the splitting of sucrose with invertase and conversion of fructose to glucose by isomerase, the sum of glucose, fructose and sucrose (referred to here as ‘sugars’) was determined photometrically at 340 nm after the conversion of glucose to gluconate‐6‐P using hexokinase (glucose assay reagent; Sigma‐Aldrich). In the remaining pellet, starch was degraded to free glucose by sequentially adding ⍺‐amylase and amyloglucosidase. The total amount of free glucose after starch degradation was determined photometrically as described above. A dilution series from a commercial glucose standard solution (1 mg ml^−1^) was used to quantify glucose. Sucrose, fructose and starch solutions, as well as two different plant powders (orchard leaves and cereal grains), were included in each analysis run to ensure correct enzyme activities and reproducibility of the analyses. All chemicals were purchased from Sigma‐Aldrich. Tissue concentrations in needles, bark and branch wood were expressed as percentages on a dry matter basis.

### Visualization of active xylem

Branches of *c*. 150 cm length, including the apical end, were cut in the morning from the upper canopies. The basal end was immediately recut under water, and the remaining branch having a length of *c*. 100 cm was brought to the laboratory with the open‐cut, basal end permanently under water. The branches were then recut at the basal end under water to a final length of *c*. 80 cm and a branch diameter at the basal end of around 0.5 cm. The branches were placed in a 0.5% aqueous solution of methylene blue (Roth AG, Arlesheim, Switzerland) and kept in a glasshouse at 25°C with additional artificial illumination. The next day, cross sections were taken in the afternoon from the middle part of the branch, rinsed with 70% ethanol and mounted with Euparal (Roth AG) on glass slides for microscopic analysis. The stained xylem area was quantified using a stereoscope (Olympus Stereomicroscope SZX7, Olympus Europa SE & CO. KG, Hamburg, Germany) equipped with a digital imaging system.

### Measurement of stomatal conductance

Stomatal conductance was measured on the same branches that were cut from the trees for visualization of active xylem using a hand‐held porometer (SC‐1 Porometer; Decagon Inc., Pullman, WA, USA). Before the measurements, the branches were kept for 1 d in the glasshouse with the basal end in the dye‐tracer solution for visualizing transpiration‐driven water transport and afterwards moved outside the glasshouse and exposed for 2 h to full natural sunlight (12:00 h to 14:00 h CEST) to avoid any light‐dependent limitations of stomatal conductance. All measurements were conducted on three selected leaves per branch in the early afternoon. The whole‐branch stomatal conductance was calculated relating average leaf stomatal conductance to total branch leaf area.

### Quantification of tree ring growth

To identify tree ring growth, semi‐thin transverse sections were obtained with a sliding microtome (G.S.L. 1; Schenkung Dapples, Zürich, Switzerland) from the samples obtained for PLC measurements. These cuts were then digitalized at ×100 magnification using a stereo microscope with an automatic stage equipped with a digital camera (Observer Z1; Carl Zeiss Microscopy GmbH, Jena, Germany) using the AxioVision software (AxioVision 4.9.1; Carl Zeiss Microscopy GmbH), and the width of recently formed tree rings was then quantified on the digital images.

### Data analysis

Data were analysed using the R statistical environment. For each response variable, a linear mixed‐effects model was fitted using health status, month (coded as factor) and their interaction as fixed effects and tree individual as random intercept, to account for repeated measurements. To ensure normality, tree ring increment was square‐root‐transformed and NSC concentration in bark tissue was log‐transformed. *Post hoc* tests with the Bonferroni correction were performed using the R package ‘emmeans’ to test differences between healthy and symptomatic trees at each measurement time. Linear regressions were calculated to test the effect of active xylem area on foliage area and of active xylem area on whole‐twig stomatal conductance. Linear regressions were calculated individually for two time points in 2019.

## Results

### Canopy health status and native loss of branch hydraulic conductance

In spring 2019, after the severe 2018 drought, we observed that many beech trees at the Swiss‐Canopy‐Crane II site and the surrounding forest area developed severe post‐drought damages in their upper canopies, with arrested buds, sparse foliation development and partial branch dieback (Fig. 2a,b). Accordingly, in the trees that we investigated for this study, the degree of foliation in upper canopy branches (ratio of leaf to sapwood area) was much lower in trees that developed such post‐drought damage symptoms than in trees remaining healthy (Fig. 2c; Tables S2, S3). Biotic agents, that is leaf‐eating insects, were not observed and could therefore be excluded as a cause of canopy defoliation.

During the 2018 summer drought, all trees showed substantial losses of branch hydraulic conductance (PLC) in their upper canopies, regardless of whether they developed the above‐described symptoms in the following year or not. In early September 2018, PLC values in single branches ranged from 30% to 80% with an average PLC across the tested individuals of 55.1% in trees remaining healthy and 50.7% in trees becoming symptomatic in 2019 (Fig. 2d; Tables S2, S3). After leaf unfolding in spring 2019 – and following the wet winter season with strongly elevated soil moisture content in between (Fig. 1) – the average PLC values in upper canopy branches of symptomatic trees increased sharply to 88.9%, while PLC values in branches from trees remaining healthy showed an opposite trend with a moderate decline to 37.0% (Fig. 2d). In the latter group, the decrease in PLC was accompanied by the formation of new xylem tissue, while branches in symptomatic trees showed no or only marginal tree ring growth in late spring 2019 (Fig. 2e; Tables S2, S3). The 2019 summer was characterized by short episodes of mild drought and heavy intermediate rainfall, lifting soil moisture temporally to levels observed during winter and spring. However, in midsummer 2019, the trees in the two categories still showed strong differences, with an average PLC of 66.2% in symptomatic trees and a further decrease to 16.9% in healthy trees. Xylem formation progressed in healthy trees but remained strongly impaired in symptomatic trees (Fig. 2e). In late summer 2019, the average PLC remained high in symptomatic trees at 69.0%, and increased again in healthy trees to 42.9%.

### Nonstructural carbohydrates in healthy and symptomatic trees

**Fig. 3:**
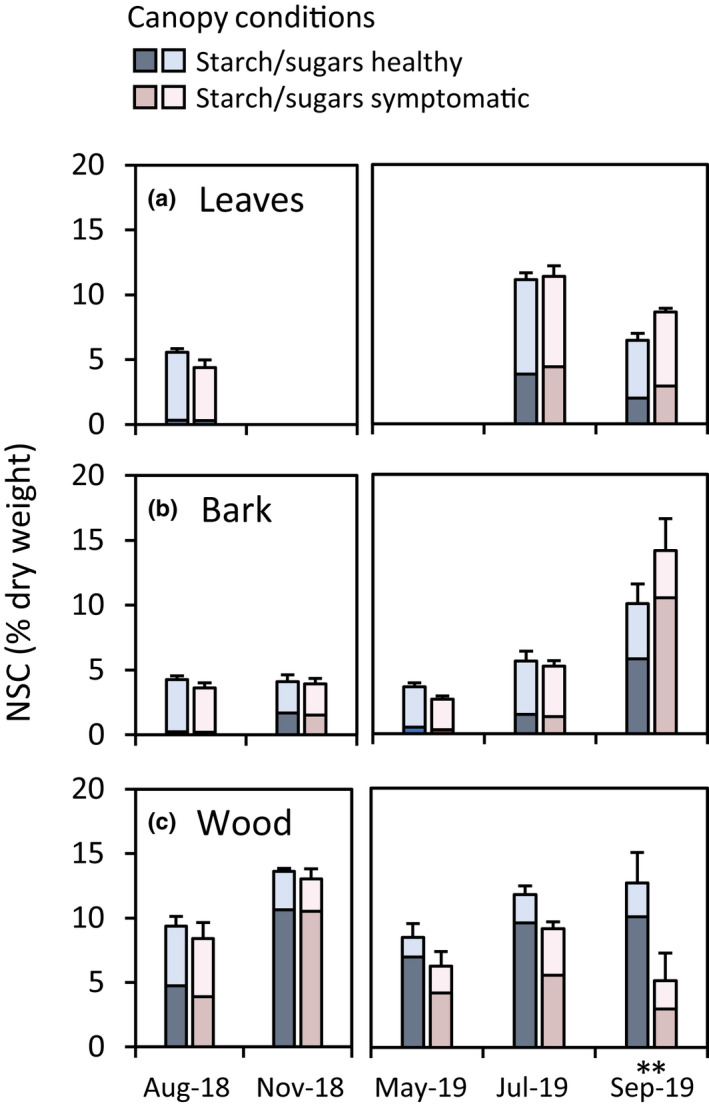
Tissue levels of nonstructural carbohydrates (NSC = sugars and starch) in upper canopy branches of beech trees during and after the severe 2018 drought. Nonstructural carbohydrate levels in (a) leaves, (b) bark and (c) wood tissue under severe drought and after drought release in 2018 (bars show the average of trees remaining healthy or becoming symptomatic in the following season 2019) and in the following summer half‐year 2019 (data are mean ± SE; *n* = 5 trees; *post hoc* tests for differences between groups with: **, *P* ≤ 0.01).

Tissue‐specific levels of nonstructural carbohydrates (NSC) were analysed during and after the 2018 drought (Fig. 3; Tables S4, S5). In summer 2018, under severe drought conditions, we found a decline of NSC reserves in upper canopy branches, manifested by nearly complete depletions of starch in bark and leaves in all trees regardless of the canopy health status in the following summer 2019. In the wood tissue, by contrast, the starch concentration remained relatively high. Importantly, starch concentrations increased quickly after drought release in autumn 2018, with no difference between trees becoming symptomatic in the next year or remaining healthy.

In spring 2019, when severe defoliation symptoms became visible in the upper parts of the beech canopies, NSC concentrations started to differ in the upper canopy branches of healthy and symptomatic trees. This was mainly due to a loss of starch in the branch wood of symptomatic trees throughout the summer, resulting in a gradual decline in NSC concentrations in symptomatic trees towards the end of summer 2019. This gradual decline in NSC concentrations was not observed in bark and leaves, where starch and NSC concentrations were nearly similar in healthy and symptomatic trees in early and midsummer, but increased in late summer in symptomatic trees compared with healthy trees (Fig. 3).

### Dye‐tracer visualization of active xylem

**Fig. 4:**
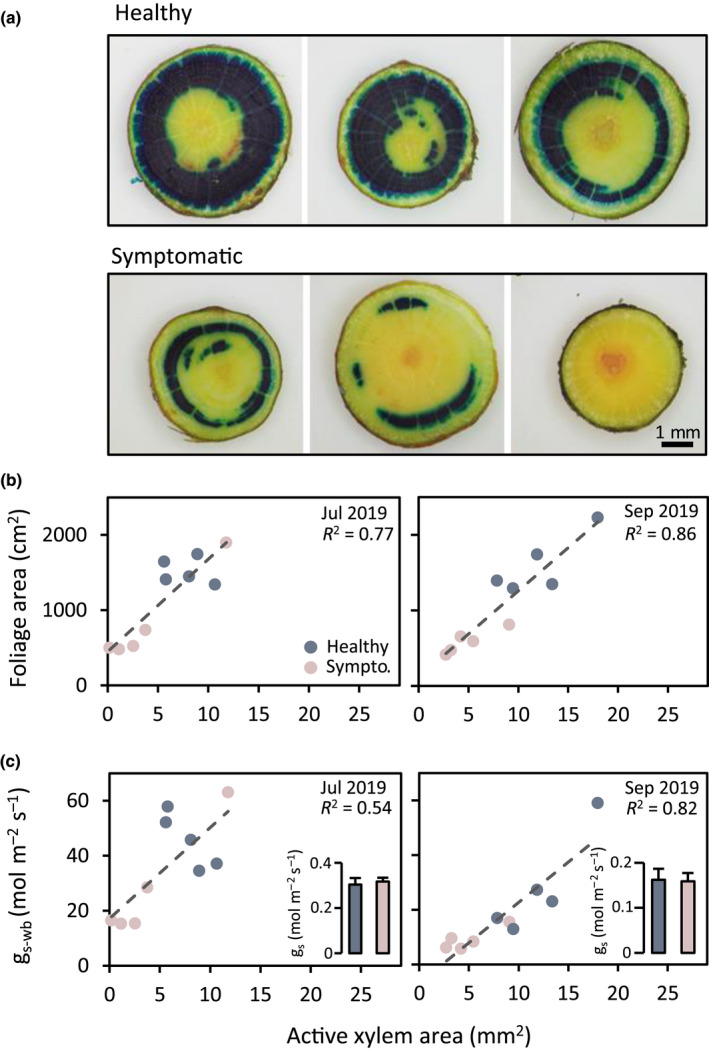
Dependency of branch foliation and whole‐branch stomatal conductance on xylem hydraulic function in beech trees in the year after the severe 2018 drought. (a) Dye‐tracer visualizations of active xylem area in cross sections of nonsymptomatic and symptomatic branches. (b) Relationships between active xylem area and branch foliation in nonsymptomatic and symptomatic branches. (c) Relationships between active xylem area and whole‐branch stomatal conductance (*g*_s‐wb_) in nonsymptomatic and symptomatic branches. Bar plots show stomatal conductance (*g*_s_) in single leaves (scattered data points represent single branches, bar plots represent means ± SE, *n* = 10).

A dye‐tracer approach was used to visualize the transpiration‐driven water flow and to quantify the active sapwood area in upper canopy branches of healthy and symptomatic trees (Fig. 4a). The axial water flow through the branch tissue was indicated by intense blue dye‐tracer staining and was exclusively confined to the xylem, though closer inspection of thinner branch cross sections revealed lateral tracer diffusion from axial xylem to the phloem and bark through radial ray parenchyma (not shown). Importantly, we often observed that some parts of the xylem remained unstained, indicating the scattered co‐occurrence of water‐conducting and embolized xylem across the branch sapwood area. Active, water‐conducting xylem was mainly found in younger tree rings and in patches of different sizes in older tree rings. Regardless of these heterogeneous staining patterns, we found strong differences in the total conducting xylem area between healthy and symptomatic branches. In general, a much larger proportion of sapwood was stained in healthy than in symptomatic branches (Fig. 4a,b), indicating that more xylem was active and contributed to axial water flow through the branch in healthy trees. In symptomatic trees, by contrast, the dye‐tracer staining was often confined to small patches in younger tree rings or was even undetectable (lower right picture in Fig. 4a).

Visualizations of active xylem in detached canopy branches were used to test relationships between reductions in the amount of active xylem area and the observed symptoms of reduced branch foliation (Fig. 4b). Branches collected in midsummer 2019 showed a strong relationship between the active xylem area and the total leaf area of a branch (*F*(1,8) = 27.29, *P* < 0.001, *R* ^2^ = 0.77; Table S6). A similar strong relationship was obtained with branches collected in late summer 2019 (*F*(1,8) = 48.56, *P* < 0.001, *R* ^2^ = 0.86; Table S6). We finally tested how the degree of branch foliation translated into whole‐branch stomatal conductance and whether similar relationships exist with the amount of active xylem as observed for the degree of branch foliation (Fig. 4c). Branches collected in midsummer 2019 showed a strong relationship between the amount of active xylem area and whole‐branch stomatal conductance (*F*(1,8) = 9.52, *P* = 0.015, *R* ^2^ = 0.54; Table S6) that got more pronounced in late summer 2019 (*F*(1,8) = 35.57, *P* < 0.001, *R* ^2^ = 0.82; Table S6). The relationship between active xylem area and whole‐branch stomatal conductance was mainly driven by the strong variations in total leaf area between healthy and symptomatic branches, while stomatal conductance per unit leaf area showed no differences between healthy and symptomatic branches (see inserts in Fig. 4c).

## Discussion

In this study on mature beech trees – conducted during and after the exceptionally severe 2018 central European drought – we obtained three important findings. First, we did not obtain any evidence for substantial embolism repair resulting in the recovery of branch hydraulic conductance over the winter and early spring following the 2018 drought. Second, symptoms of reduced branch foliation occurring in the year after the 2018 drought were associated with persisting, and in some trees, further progressing, branch hydraulic dysfunction. Hence, trees becoming symptomatic in 2019 showed much stronger reductions in branch hydraulic conductance than trees remaining healthy and the lack of hydraulic recovery in those trees was accompanied by strongly diminished wood growth. Third, dye‐tracer studies on detached branches revealed a strong relationship between the amount of active xylem and the degree of branch foliation, indicating a strong effect of xylem hydraulic damage on post‐drought leaf development. These findings may not only explain widespread symptoms of post‐drought canopy decline in European beech but may also provide empirical evidence for sustained hydraulic dysfunction in mature trees in the growing season following a severe drought.

A question that is central to the above findings and that overarches any mechanistic approach to drought‐induced forest decline is, why do mature forest trees enter different pathways of recovery after a severe drought? Some uncertainty is attributable to the overall difficulties in studying mechanisms of drought resistance and recovery in tall trees, in which the underlying functions may differ in distal and basal tree organs (Choat *et al*., 2005). Another explanation relates to the simple fact that a mature tree develops individual root and crown characteristics over its long lifetime and grows in a heterogeneous environment, where spatial variations of accessible soil water and different exposures to the free atmosphere are common. All this may affect a tree’s internal water balance, leading to different intensities of hydraulic stress that individual trees experience during a drought. This in turn may interfere with the variation of xylem hydraulic vulnerability among and within trees that is well documented for beech and other species (Wortemann *et al*., 2011; Hajek *et al*., 2016; Schuldt *et al*., 2016; Cardoso *et al*., 2020; Lübbe *et al*., 2022). All these sources of biological and environmental variation were apparent in our study, which may well explain why some trees experienced of persisting canopy dysfunction after the severe 2018 drought while others did not.

The principal physiological mechanism that constrains a tree’s ability to recover from drought‐induced damage concerns its ability to restore losses of xylem hydraulic conductance. There are two conceivable pathways for the recovery: repair of embolized xylem after stress release (Zwieniecki & Holbrook, 2009; Nardini *et al*., 2011; Brodersen & McElrone, 2013) and growth of new functional xylem (Ameglio *et al*., 2002; Brodribb *et al*., 2010; Trugman *et al*., 2018). While it is obvious that xylem growth must eventually occur in surviving trees that recovered, the significance and physiological nature of embolism repair is still debated (Klein *et al*., 2018). Basic work in the field of embolism repair has been done on detached branches with water potentials close to zero (Borghetti *et al*., 1991; Earles *et al*., 2016; Knipfer *et al*., 2016; Trifilò *et al*., 2019), conditions that hardly reflect the situation in intact trees after a drought. On the contrary, studies on intact trees provided controversial results, either supporting or questioning embolism repair in trees (McCulloh *et al*., 2011; Choat *et al*., 2015; Savi *et al*., 2016; Tomasella *et al*., 2017; Li *et al*., 2018; Rehschuh *et al*., 2020). Thus, it remains unclear whether it is a universal phenomenon in nature, and if so, how it actually contributes to the restoration of tree hydraulic function.

In our study, we did not obtain evidence for embolism repair, as PLC remained high at the beginning of the summer following the 2018 drought and even further increased in trees developing symptoms of canopy decline. In trees remaining healthy, we observed a decline in PLC with progressing xylem formation, before it increased again at the end of the summer. In contrast to healthy trees, xylem growth was negligible in symptomatic trees with high PLC. This suggests that post‐drought xylem growth and not embolism repair is the major process driving hydraulic recovery in mature trees that have experienced a severe drought. It is noteworthy that hydraulic recovery may not only be constrained by the limitation of post‐drought xylem growth but also by concomitant anatomical adjustments of newly formed xylem conduits (Islam *et al*., 2018).

Post‐drought xylem growth is central to recent frameworks conceptualizing mechanisms of tree hydraulic recovery and has been exclusively linked to the maintenance of a positive whole‐tree carbon gain after drought (Trugman *et al*., 2018). Conversely, negative carbon gains and depletion of carbohydrate reserves have been proposed as major causes of diminished post‐drought xylem growth and persisting xylem hydraulic dysfunction. Our observations support the hypothesis of xylem growth as a central mechanism for the restoration of hydraulic conductance. However, our observations do not support the assumption that the lack of xylem formation after drought is a consequence of low carbohydrate availability as post‐drought NSC concentrations in our study showed relatively small and delayed reductions in symptomatic trees suffering from high loss of xylem hydraulic conductance. Instead, sparse foliage development might have played a particular role. In fact, it is generally accepted that the production of growth‐stimulating auxins and other hormonal growth regulators in outgrowing buds and young leaves is required for activation of xylem growth, and debudding or defoliation has been shown to inhibit the growth of new xylem (Little & Savidge, 1987; Uggla *et al*., 1996; Sundberg *et al*., 2000; Arend *et al*., 2002; Schmid *et al*., 2017). Importantly, a possible disruption of the hormonal control of xylogenesis following drought has not received attention in current approaches explaining tree hydraulic recovery.

Current plant hydraulic theory predicts serious consequences of hydraulic failure for the functional integrity of trees and tree organs (Adams *et al*., 2017). The canopy decline symptoms in beech trees, which failed to restore branch hydraulic conductance in the season following the 2018 drought and suffered from further loss of branch hydraulic conductance, are in line with this theory. It complements previous studies suggesting hydraulic failure as a major cause of drought‐induced canopy dieback and mortality in European beech and other tree species (Barigah *et al*., 2013; Li *et al*., 2018; Hajek *et al*., 2020; Arend *et al*., 2021; Nolan *et al*., 2021; Walthert *et al*., 2021). However, we also show that canopy decline in response to hydraulic failure is not necessarily an immediate drought response but may occur in seasons following a drought. The reason why symptomatic trees showed even higher losses of branch hydraulic conductance in the wet spring 2019 compared with the preceding measurements in the dry summer 2018 remains an open question. Yet, we cannot exclude further embolism formation after the measurements in summer 2018, as sustained soil rewetting did not occur before the end of the drought in autumn. It is unlikely that carbohydrate depletion triggered this process, as the NSC concentrations did not differ after stress release in 2018 in trees remaining healthy or becoming symptomatic.

The observed limitations of hydraulic function and foliage development undoubtedly have serious consequences for trees. Importantly, reduced foliation and partial branch dieback may also serve as a post‐drought acclimation response that rebalances root water uptake and transpirational water loss through canopies over longer developmental timescales (Rood *et al*., 2000; Bréda *et al*., 2006; Trugman *et al*., 2018; Pritzkow *et al*., 2021). This is in good agreement with recent studies linking observations of heterogeneous leaf or branch mortality across the canopy of drought‐stressed trees with hydraulic dysfunction, and based on this, partial leaf or branch mortality has been proposed to act as buffer against complete dehydration and tree death during prolonged drought (Davis *et al*., 2002; Cardoso *et al*., 2020). Similarly, the persisting loss of branch hydraulic conductance and associated leaf area reduction in upper canopies of symptomatic beech trees might help with reorganizing their hydraulic structure to reduce excessive water loss and thus prepare the trees for recurrent droughts. It remains, however, to be shown whether a partial loss of the transpiring canopy leaf area actually serves as an acclimation response that improves a tree’s resistance to recurrent droughts.

Regardless of the fate of symptomatic beech trees under future droughts, we show that they counterbalance impaired branch xylem function with reduced foliage development, which ultimately leads to reductions in canopy water demand. This is intriguingly demonstrated by the close relationship between the functional xylem area in branch cross sections and the degree of branch foliation or whole‐branch stomatal conductance that we observed in dye‐tracer studies on detached branches. Notably, we can exclude adjustments on the stomatal level, as single leaf stomatal conductance remained unaffected across healthy and symptomatic trees. These observations complement current knowledge on allometric sapwood‐to‐leaf area relationships, as expressed in the classical Huber value (Huber, 1928; Tyree & Ewers, 1991), by a stress–physiological component. Importantly, we show that branch foliation may respond to drought‐induced damages of hydraulic sapwood function at much shorter timescales than conservative definitions of the Huber value may allow. In fact, in its strict sense, the Huber value is thought to be a species‐specific allometric trait that guarantees the long‐term development of the transpiring canopy is in functional equilibrium with the water‐transporting sapwood area under average environmental conditions (Mencuccini *et al*., 2019). Yet, studies on several woody species in semi‐dry areas have shown that the Huber value may vary seasonally as a result of drought‐induced leaf shedding (O'Grady *et al*., 2009). Xylem hydraulic failures may further compromise the functional equilibrium of sapwood with distal leaf organs, if not counterbalanced by subsequent leaf area reductions, as shown in this study.

Even though the Huber value in its classical sense is a fundamental developmental trait that describes the relative carbon costs of xylem and leaf hydraulic construction, the above considerations may demonstrate the limits of its stress–physiological interpretation under the conditions of extreme stress. Independent of the developmental borders that the classical definition of the Huber value set, we show a tight coordination of inter‐seasonal leaf development with altered hydraulic sapwood function that keeps branch foliation in close equilibrium with the actual capacity of the branch to transport water. Similar relationships have been found in defoliation experiments, where artificial leaf removal resulted in concordant reductions in tree ring growth (Schmid *et al*., 2017). This and our own observations point to a high adaptability of the branch hydraulic system to drought‐induced damages. Finally, it does not only provide a physiological explanation of the observed post‐drought canopy damage in beech but also supports recent views that persisting limitations of tree function after a drought share similar mechanisms with processes conferring acclimation to recurrent drought (Gessler *et al*., 2020). In this sense, post‐drought reductions in transpiring canopy leaf area may potentially increase the resistance of the tree hydraulic system to recurrent droughts, yet the significance of post‐drought canopy reductions as acclimation response needs to be tested in future studies.

## Author contributions

MA designed the study. RML and BS measured native loss of xylem hydraulic conductance. MA performed the dye‐tracer experiment and measured stomatal conductance. GH, CZ and MA analysed nonstructural carbohydrates. MA, CZ and RML analysed the data. MA and AK wrote the manuscript with contributions from BS, GH and RML.

## Supporting information


**Table S1** Minimum xylem pressures, and xylem pressures at 12%, 50% and 88% loss of hydraulic conductance.
**Table S2** Analysis of deviance tables for branch foliation, loss of hydraulic conductance and tree ring increment.
**Table S3** *Post hoc* tests for branch foliation, loss of xylem hydraulic conductance and tree ring increment.
**Table S4** Analysis of deviances for non‐structural carbohydrate concentrations.
**Table S5** *Post hoc* tests for non‐structural carbohydrate concentrations.
**Table S6** Linear regression for foliage area on active xylem area.
Please note: Wiley Blackwell are not responsible for the content or functionality of any Supporting Information supplied by the authors. Any queries (other than missing material) should be directed to the *New Phytologist* Central Office.

## Data Availability

All data are included in the manuscript and Supporting Information. Raw data are available upon request.
