# Supplementary material for: Lack of hydraulic recovery as a cause of post‐drought foliage reduction and canopy decline in European beech
Source: New Phytol. 2022 Mar 23;234(4):1195–205. doi: 10.1111/nph.18065 (PMC9310744; doi:10.1111/nph.18065)
Supplement: Supplementary file 1 — Table S1 Minimum xylem pressures, and xylem pressures at 12%, 50% and 88% loss of hydraulic conductance. Table S2 Analysis of deviance tables for branch foliation, loss of hydraulic conductance and tree ring increment. Table S3 Post hoc tests for branch foliation, loss of xylem hydraulic conductance and tree ring increment. Table S4 Analysis of deviances for non‐structural carbohydrate concentrations. Table S5 Post hoc tests for non‐structural carbohydrate concentrations. Table S6 Linear regression for foliage area on active xylem area. Please note: Wiley Blackwell are not responsible for the content or functionality of any Supporting Information supplied by the authors. Any queries (other than missing material) should be directed to the New Phytologist Central Office. [file NPH-234-1195-s001.pdf]

New Phytologist **Supporting Information**

Article title: Lack of hydraulic recovery as cause of post-drought foliage reduction and canopy decline in European beech
Authors: Matthias Arend, Roman Mathias Link, Cedric Zahnd, Günter Hoch, Bernhard Schuldt, Ansgar Kahmen
Article acceptance date: 19 February 2022

The following Supporting Information is available for this article:

Table S1 Minimum xylem pressures, and xylem pressures at 12%, 50% and 88% loss of hydraulic conductivity

Range of minimum xylem pressures (P_{min}) in beech trees at the Swiss-Canopy-Crane II site during the 2018 drought and xylem pressures at 12%, 50% and 88% loss of hydraulic conductivity (P_{12}, P_{50}, P_{88}) obtained from vulnerability curves with independently selected trees on the same site. Minimum xylem pressures were measured at midday using a pressure chamber according to Arend et al., 2021. The flow-centrifuge technique (Cochard et al., 2005) was used to construct xylem vulnerability curves for one branch from the uppermost canopy from nine trees sampled in October 2019 according to a standard protocol (for details, please refer to Schuldt et al., 2016).

	P_{min} (MPa)	P_{12} (MPa)	P_{50} (MPa)	P_{88} (MPa)
mean ± SD	-2.7 ± 0.3	-2.7 ± 0.2	-3.4 ± 0.2	-4.0 ± 0.3
minimum	-3.3	-3.0	-3.5	-4.4
maximum	-2.4	-2.5	-3.1	-3.6
n	11	9	9	9

Table S2 Analysis of deviance tables for branch foliation, loss of hydraulic conductance and tree ring increment

Analysis of deviance tables (type II tests) for degree of branch foliation (LA:SA, total leaf area normalized to sap wood area), native loss of hydraulic conductance (PLC) and tree ring increment (TRI), each by health status (healthy vs. symptomatic), month and their interaction (see main text figure 2c - e). All models included tree individual as random intercept. TRI was square root transformed to ensure normality.

Response	Predictor	χ^2	df	p
LA:SA	Health status	109.644	1	<0.001
	Month	1.559	2	0.459
	Health status : month	6.751	2	0.034
PLC	Health status	14.137	1	<0.001
	Month	20.919	3	<0.001
	Health status : month	23.455	3	<0.001
TRI	Health status	96.188	1	<0.001
	Month	66.225	2	<0.001
	Health status : month	6.558	2	0.038

Table S3 Post-hoc tests for branch foliation, loss of xylem hydraulic conductance and tree ring increment

Post-hoc tests for symptomatic – healthy trees for each month based on the respective models given in Tab. S2, for degree of branch foliation (LA:SA), native loss of hydraulic conductance (PLC) and tree ring increment (TRI). All P-values were Bonferroni corrected. Estimates for TRI are back-transformed.

Response	month	estimate	SE	df	t ratio	p
	Jun 19	-0.406	0.048	8	-0.094	<0.001
LA:SA	Jul 19	-0.325	0.048	8	-7.279	<0.001
	Sep 19	-0.265	0.048	8	-5.938	0.001
	Sep 18	4.13	9.76	13	0.423	1.000
PLC	Jun 19	50.93	9.96	13	5.115	<0.001
	Jul 19	48.15	9.96	13	4.835	0.001
	Sep 19	25.24	9.96	13	2.535	0.100
	Jun 19	-0.095	0.019	9	-4.995	0.002
TRI	Jul 19	-0.258	0.033	9	-7.788	<0.001
	Sep 19	-0.230	0.033	9	-7.063	<0.001

Table S4 Analysis of deviances for NSC concentration

Analysis of deviance tables (type II tests) for total non-structural carbohydrates (NSC) concentrations in leaves, bark and wood, each by health status (healthy vs. symptomatic), month and their interaction (see main text figure 3a - c). All models included tree individual as random intercept. Bark NSC concentration was log transformed to ensure normality.

**Response (Tissue)**	**Predictor**	χ^2	**df**	**p**
Leaves	Health status	0.596	1	0.440
	Month	96.301	2	<0.001
	Health status : month	7.203	2	0.027
Bark	Health status	0.003	1	0.960
	Month	122.271	4	<0.001
	Health status : month	6.293	4	0.178
Wood	Health status	4.399	1	0.036
	Month	32.079	4	<0.001
	Health status : month	13.571	4	0.009

Table S5 Post-hoc tests for NSC concentrations

Post-hoc tests for symptomatic – healthy trees for each month based on the respective models given in Tab. S4, for total non-structural carbohydrate (NSC) concentrations in leaves, bark and wood. All P-values were Bonferroni corrected. Estimates for bark NSC are back-transformed.

Response (Tissue)	month	estimate	SE	df	t ratio	p
Leaves	Aug 18	-1.176	0.880	9	-1.336	0.643
	Jul 19	0.248	0.934	9	0.266	1.000
	Sep 19	2.190	0.898	9	2.438	0.113
Bark	Aug 18	0.199	0.791	15	0.252	1.000
	Nov 18	0.099	0.724	15	0.137	1.000
	May 19	-0.979	0.619	15	-1.582	0.672
	Jul 19	-0.414	0.994	15	-0.417	1.000
	Sep 19	3.305	2.193	15	1.507	0.763
Wood	Aug 18	0.201	1.89	20	0.106	1.000
	Nov 18	0.006	1.96	20	0.003	1.000
	May 19	-2.415	1.97	20	-1.227	1.000
	Jul 19	-2.760	1.97	20	-0.401	0.883
	Sep 19	-7.758	1.96	20	-3.956	0.004

Table S6 Linear regression for foliage area on active xylem area

Linear regression parameters for branch foliage area on active xylem area in July 2019 and September 2019 (see main text figure 4b), and for whole branch stomatal conductance ($g_{s\text{-}wb}$) on active xylem area in July 2019 and September 2019 (see main text figure 4c). (For F statistics and R^2 values see results section in the main text)

Response	Variable	Estimate	SE	t	p
Foliage area – Jul. 19	Intercept	450.5	163.9	2.748	0.025
	Slope	122.8	23.5	5.224	<0.001
Foliage area – Sep. 19	Intercept	114.5	159.6	0.718	0.493
	Slope	114.3	16.4	6.969	<0.001
$g_{s\text{-}wb}$ – Jul. 19	Intercept	17.2	7.5	2.299	0.051
	Slope	3.3	1.1	3.085	0.015
$g_{s\text{-}wb}$ – Sep. 19	Intercept	-6.8	4.8	-1.409	0.197
	Slope	2.9	0.5	5.964	<0.001

References

Arend M, Schuldt B, Link RM, Patthey R, Hoch G, Kahmen A. 2021. Rapid hydraulic collapse as cause of drought-induced mortality in conifers. *Proceedings of the National Academy of Sciences USA* **118:** e2025251118, doi: 10.1073/pnas.2025251118

Cochard H, Damour G, Bodet C, Tharwat I, Poirier M, Améglio T. 2005. Evaluation of a new centrifuge technique for rapid generation of xylem vulnerability curves. *Physiologia Plantarum* **124:** 410-418. https://doi.org/10.1111/j.1399-3054.2005.00526.x

Schuldt B, Knutzen F, Delzon S, Jansen S, Müller-Haubold H, Burlett R, Clough Y, Leuschner C. 2016. How adaptable is the hydraulic system of European beech in the face of climate change-related precipitation reduction? *New Phytologist* **210:** 443–458.
